# Intensity modulated radiation therapy versus three‐dimensional conformal radiation therapy for the treatment of high grade glioma: a dosimetric comparison

**DOI:** 10.1120/jacmp.v8i2.2423

**Published:** 2007-04-19

**Authors:** Shannon M. MacDonald, Salahuddin Ahmad, Stefanos Kachris, Betty J. Vogds, Melissa DeRouen, Alicia E. Gitttleman, Keith DeWyngaert, Maria T. Vlachaki

**Affiliations:** ^1^ Massachusetts General Hospital Boston Massachusetts; ^2^ University of Oklahoma Health Sciences Center Oklahoma City Oklahoma; ^3^ New York University Medical Center New York New York; ^4^ Wayne State University Detroit Michigan U.S.A.

**Keywords:** high‐grade glioma, IMRT, 3D‐CRT

## Abstract

The present study compared the dosimetry of intensity‐modulated radiation therapy (IMRT) and three‐dimensional conformal radiation therapy (3D‐CRT) techniques in patients treated for high‐grade glioma. A total of 20 patients underwent computed tomography treatment planning in conjunction with magnetic resonance imaging fusion. Prescription dose and normal‐tissue constraints were identical for the 3D‐CRT and IMRT plans. The prescribed dose was 59.4 Gy delivered at 1.8 Gy per fraction using 4 – 10 MV photons. Normal‐tissue dose constraints were 50 – 54 Gy for the optic chiasm and nerves, and 55 – 60 Gy for the brainstem.

The IMRT plan yielded superior target coverage as compared with the 3D‐CRT plan. Specifically, minimum and mean planning target volume cone down doses were 54.52 Gy and 61.74 Gy for IMRT and 50.56 Gy and 60.06 Gy for 3D‐CRT (p≤0.01). The IMRT plan reduced the percent volume of brainstem receiving a dose greater than 45 Gy by 31% (p=0.004) and the percent volume of brain receiving a dose greater than 18 Gy, 24 Gy, and 45 Gy by 10% (p=0.059), 14% (p=0.015), and 40% (p≤0.0001) respectively. With IMRT, the percent volume of optic chiasm receiving more than 45 Gy was also reduced by 30.40% (p=0.047). As compared with 3D‐CRT, IMRT significantly increased the tumor control probability (p≤0.005) and lowered the normal‐tissue complication probability for brain and brainstem (p<0.033).

Intensity‐modulated radiation therapy improved target coverage and reduced radiation dose to the brain, brainstem, and optic chiasm. With the availability of new cancer imaging tools and more effective systemic agents, IMRT may be used to intensify tumor doses while minimizing toxicity, therefore potentially improving outcomes in patients with high‐grade glioma.

PACs number: 87.53 Tf

## I. INTRODUCTION

Few malignancies are considered more devastating or challenging to control than malignant glioma. With standard therapy consisting of maximal surgical resection followed by involved‐field radiation and chemotherapy, the median and 2‐year survival for glioblastoma multiforme are 12 months and 10% respectively.^(^ ^1^ ^,^ ^2^ ^)^ Recent advances in chemotherapy have increased survival in glioblastoma, especially for patients with favorable prognostic factors.^(^ ^3^ ^–^ ^5^ ^)^ Unfortunately, these patients are at high risk of late radiation toxicity, including radiation necrosis and neurocognitive deficits.^(3)^ As the number of long‐term survivors increases, an increase will almost certainly be seen in the number of patients suffering from the late effects of radiation. Therefore, to ensure optimal tumor coverage with minimal radiation injury, investigating the integration of advanced, highly conformal radiotherapy techniques for this disease is of clinical importance.

Local recurrences are by far the most frequent cause of treatment failure in patients with high‐grade glioma.^(^ ^6^ ^,^ ^7^ ^)^ In the 1970s, Walker and colleagues from the Brain Tumor Study Group discovered a radiation dose–response relationship, and a dose of 60 Gy was established as the standard of care.^(8)^ Further dose intensification through higher radiation doses and altered fractionation was pursued, but failed to provide a clear clinical benefit.^(^ ^9^ ^,^ ^10^ ^)^ Currently, 60 Gy remains the standard dose for high‐grade glioma. However, with advances in diagnostic imaging and radiotherapy techniques, the role of radiation dose intensification in the management of high‐grade glioma is likely to undergo further exploration.

Past studies indicated that chemotherapy could provide a modest survival benefit in patients with high‐grade glioma.^(^ ^6^ ^,^ ^11^ ^,^ ^12^ ^)^ However, a recent phase III randomized trial by Stupp et al.^(5)^ demonstrated a 2‐year survival increase from 12.9% with radiation alone to 26% with the use of concomitant and adjuvant temozolomide. Further molecular analysis showed a 46% 2‐year survival in a favorable subset of patients with epigenetic silencing of the O^6^‐methylguanine‐DNA methyltransferase DNA‐repair gene.^(^ ^13^ ^–^ ^15^ ^)^

As knowledge of cancer genomics and proteomics evolves, new, promising agents that target specific molecular aberrancies in individual brain tumors are being developed. These targeted agents have already produced favorable responses in the setting of progressive or recurrent disease, and they are currently being tested as first‐line therapy in high‐grade glioma.^(^ ^16^ ^–^ ^18^ ^)^

It is evident that advances in the diagnosis and management of high‐grade glioma will further increase the number of long‐term survivors of this disease. Consequently, the risk of radiation‐related late effects will undoubtedly rise. That situation and the overwhelming incidence of local failure with current treatment are certainly encouraging further attempts to improve radiation dose delivery.

Intensity‐modulated radiation therapy (IMRT) has been proven to optimize target dose and to simultaneously decrease the dose to normal structures. It has improved outcomes in several malignancies.^(^ ^19^ ^–^ ^21^ ^)^ However, data regarding the use of IMRT for high‐grade glioma are limited.^(^ ^22^ ^,^ ^23^ ^)^ In the present study, we compared dosimetry for IMRT and three‐dimensional conformal radiation therapy (3D‐CRT) techniques in 20 patients with high‐grade glioma. Comparisons of tumor control probability (TCP) and normal‐tissue complication probability (NTCP) are also reported, and the impact of clonogen cell density (CCD) on TCP is discussed. Finally, the potential for using IMRT to intensify local therapy by means of radiation dose escalation or integration of radiation therapy with novel diagnostic and therapeutic tools is discussed.

## II. MATERIALS AND METHODS

We compared IMRT and 3D‐CRT radiation treatment plans for 20 patients with high‐grade glioma treated at New York University Medical Center. In 11 patients, the tumor involved the frontal or parietal lobe (or both), and in 9 patients, it involved the temporal or occipital lobe (or both). Institutional review board approval was obtained before the review.

### A. Computed tomography planning and immobilization

A dedicated planning computed tomography (CT) scan was obtained for each patient. Patients were simulated in the supine position with head placed in a neutral to flexed position using a Duncan headrest (MJ Equipment, Inner Grove Heights, MN). Immobilization was achieved by using an Aquaplast facemask (WFR Aquaplast, Wyckoff, NJ). Axial images in 1.25‐mm increments were obtained. A separate magnetic resonance image (MRI) was obtained for each patient at diagnosis and within 48 hours after surgical resection. The T1 post‐gadolinium and T2 fluid‐attenuated inversion recovery (flair) sequences were anatomically registered to the planning CT scan using the Varian Eclipse treatment planning system (External Beam Planning 6.5, version 7.3.10: Varian Medical Systems, Palo Alto, CA) to facilitate accurate volume definition.

### B. Tumor delineation

In most of the cases, the preoperative MRI was used to delineate target volumes. The exceptions were the cases in which significant shifting of brain tissue occurred as a result of tumor resection. In those cases, the postoperative MRI was used in conjunction with information obtained from the preoperative images. The planning tumor volume (PTV) was defined as the T2 flair area of involvement with a 2‐cm margin. The cone down volume $\left({\text{PTV}}_{\text{cd}}\right)$ was defined as the T1 area of gadolinium enhancement with a 1.5‐cm to 2‐cm margin. Adjustments in the tumor volumes for bony structures and body contour were made at the discretion of the treating physician.

### C. Prescription and treatment planning

The beam arrangement was determined by the size and location of the tumor. For 3D‐CRT plans, we used two or three wedge‐paired fields by combining beams of anteroposterior or posteroanterior direction with lateral beams. For IMRT planning, we used four or five fields, also including oblique beams. Photon energies of 4 MV or a combination of 4 MV and 10 MV were used for the 3D‐CRT and IMRT plans alike. Non‐co‐planar fields were also used to optimize target dosing in some cases.

Prescription dose and normal‐tissue constraints were identical for the 3D‐CRT and IMRT plans. The prescription dose to the PTV was 45 Gy. An additional dose of 14.4 Gy was prescribed to the ${\text{PTV}}_{\text{cd}}$ to bring the total ${\text{PTV}}_{\text{cd}}$ dose to 59.4 Gy. Plans were acceptable when the 95% isodose line covered 95% of the ${\text{PTV}}_{\text{cd}}$.

The normal tissues contoured included the optic nerves, optic chiasm, left cochlea, right cochlea, brainstem, and whole brain. “Brain” was defined as total brain tissue minus the PTV. Normal‐tissue dose constraints were 50 – 54 Gy for the optic nerves and optic chiasm, and 55 – 60 Gy for the brainstem.

Tumor and normal‐tissue dose–volume histograms were generated for each plan. The IMRT and 3D‐CRT plans were then compared. All dose calculations and IMRT fluence optimizations were performed using the Varian Eclipse treatment planning system.

### D. Biologic response models

Biologic response models of TCPs and NTCPs were calculated and compared for the IMRT and 3D‐CRT plans.^(^ ^24^ ^–^ ^35^ ^)^

#### *D.1 TCP*

The quantitative biophysical measure of tumor dose, TCP, has been estimated using the numerically calculated equivalent uniform dose (EUD) obtained from the dose distributions in the target volume.^(24)^ The concept of EUD assumes that two different target dose distributions are equivalent if they cause the same radiobiologic effect, such that the corresponding number of expected surviving clonogens is equal.

Most mechanistic TCP models proceed from the hypothesis that local control of a tumor is achieved when all the clonogenic cells are destroyed by radiation because, theoretically, only 1 clonogenic tumor cell is required to repopulate the tumor. The random killing of clonogens by irradiation is well described by Poisson statistics.^(^ ^33^ ^,^ ^34^ ^)^ Using the linear quadratic expression for cell killing, we calculated TCP with the following formula:

(1) $$\text{TCP}=e^{-N{\left(SF_{2}\right)}^{\frac{D}{D_{ref}}\;\cdot \;\frac{\alpha /\beta +D/n}{\alpha /\beta +D_{ref}}}},$$

where *N* is the number of clonogen tumor cells, calculated by multiplying the CCD by the ${\text{PTV}}_{\text{cd}}$, which ranged from 18.9 cm^3^ to 383.5 cm^3^ for the 20 patients in this study; α/β=10 Gy, α and β being radiosensitivity parameters related to cell killing from single‐hit or multiple‐hit events respectively; and $SF_{2}$ is the surviving fraction after irradiation at a reference dose $\left(D_{\text{ref}}\right)$ of 2 Gy. In the present work, we assumed that 50% of clonogenic cells survive after each irradiation fraction, and therefore, $SF_{2}=0.5$; *D* equals the EUD (in Gy); and *n* is the number of treatment fractions.

The CCD is expressed in millions of tumor cells (M) per cubic centimeter (cm^3^) of irradiated tumor volume $\left({\text{PTV}}_{\text{cd}}\right)$, and its value varies from patient to patient depending on the extent of surgical tumor resection. In addition, CCD may also vary within the ${\text{PTV}}_{\text{cd}}$, because that volume consists of both gross tumor and microscopic disease. Because the true CCD value for each patient is not known, we calculated the TCP using progressively increasing CCDs from $0.1\;M/{\text{cm}}^{3}$ to $10\;M/{\text{cm}}^{3}$.

#### *D.2 NTCP*

With the treatment of tumors, normal tissues receive a radiation dose, and the NTCP is the probability that a certain percentage of the patient population will incur unfavorable reactions in the contiguous tissue at a particular dose. We calculated the NTCP of an organ for a uniform dose *D* to a volume *V* using the Lyman—Kutcher—Burman method,^(^ ^28^ ^–^ ^30^ ^)^ which is given by

(2) $$NTCP=\frac{1}{\sqrt{2\pi }}\int_{-\infty }^{t}e^{-t^{2/2}}\cdot dt,$$

where

(3) $$t=\frac{D-TD_{50}(V)}{m\cdot TD_{50}(V)}\;.$$

The $TD_{50}(V)$ is the tolerance dose for a 50% probability of complication attributable to uniform irradiation of a partial volume *V*. That dose is related to the tolerance dose for the whole organ, $TD_{50}$(1), by

(4) $$TD_{50}(V)=\frac{TD_{50}(1)}{{\left(V\right)}^{n}}\;\;.$$

In the foregoing equations, parameters *m* and *n* respectively deal with slope of the dose–response curve and the volume effect. These parameters and the $TD_{50}$(1) were obtained from listed data by Burman et al. and are shown in Table 1.^(30)^

**Table 1:** Normal‐tissue tolerance parameters for calculation of normal‐tissue complication probability

Organ at risk	$TD_{50}$(1) (Gy)	*n*	*m*
Brainstem	65	0.16	0.14
Brain–PTV	60	0.25	0.15
Optic nerves	65	0.25	0.14
Optic chiasm	65	0.25	0.14
Cochlea	60	0.25	0.15

$TD_{50}(1)=$ tolerance dose to the organ; n=volume effect; m= slope of the dose–response curve; PTV=planning tumor volume.

## III. RESULTS

As compared with the 3D‐CRT plans, the IMRT plans provided superior target coverage. With IMRT, the mean, minimum, and maximum PTV doses were 58.71, 41.75, and 63.45 Gy respectively; for 3D‐CRT, they were 57.85, 38.42, and 62.80 Gy. The differences were statistically significant for the mean and maximum doses (p≤0.023). The mean, minimum, and maximum ${\text{PTV}}_{\text{cd}}$ doses were, respectively, 61.74, 54.52, and 63.44 Gy for IMRT, and 60.06, 50.56, and 62.58 Gy for 3D‐CRT (p≤0.01). Also, we observed a significant reduction in the percent ${\text{PTV}}_{\text{cd}}$ receiving less than the prescribed dose of 59.4 Gy with IMRT (3.30% vs. 22.59% with 3D‐CRT, p≤0.0001).

Table 2 lists the ${\text{PTV}}_{\text{cd}}$ mean doses and EUDs for all 20 patients. The EUD is a surrogate of the mean dose, but it also accounts for target dose inhomogeneity. The EUD was found to be significantly lower for 3D‐CRT plans (58.06 Gy) than for IMRT plans (60.76 Gy, p≤0.0001).

**Figure 1:**
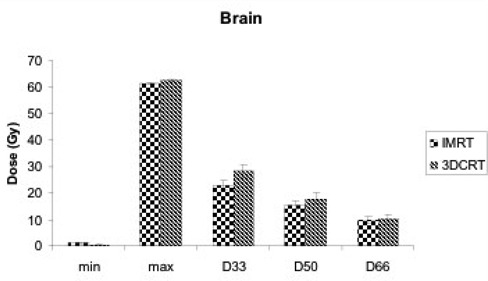
Minimum dose (min), maximum dose (max), and dose to 33% of organ volume ${(D}_{33})$, 50% of organ volume ${(D}_{50})$, and 66% of organ volume ${(D}_{66})$ for normal brain with intensity‐modulated radiation therapy (IMRT) and three‐dimensional conformal radiation therapy (3D‐CRT) plans.

The IMRT plans also provided significantly reduced doses to brain, brainstem, and optic chiasm than the 3D‐CRT plans did. Fig. 1 demonstrates that, for brain (defined as “brain minus PTV”), the doses to 33% $\left(D_{33}\right)$, 50% $\left(D_{50}\right)$, and 66% $\left(D_{66}\right)$ of the organ volume were lower with IMRT (22.96, 15.40, and 9.81 Gy respectively) than with 3D‐CRT (28.48, 17.86, and 10.16 Gy). These differences were statistically significant for $D_{33}\;(p=0.014)$. The same trend was noted in the $D_{33}$, $D_{50}$, and $D_{66}$ for brainstem. Specifically, $D_{33}$, $D_{50}$, and $D_{66}$ were 34.21, 27, and 21.41 Gy respectively for IMRT and 36.83, 29.95, and 23.14 Gy for 3D‐CRT.

For all 20 patients, Table 3 compares the percent brain volumes receiving more than 18, 24, and 45 Gy with the IMRT and 3D‐CRT plans. As compared with 3D‐CRT, IMRT reduced the percent brain volume receiving more than 18, 24, and 45 Gy by 10% (p=0.059), 14% (p=0.015), and 40% (p<0.0001) respectively. Table 4 demonstrates that IMRT also significantly reduces the brainstem volumes receiving more than 45 Gy by 31% (p=0.004).

**Table 2:** Planning tumor cone down volume $\left({\text{PTV}}_{\text{cd}}\right)$ mean and equivalent uniform doses (EUDs) delivered by intensity‐modulated radiation therapy (IMRT) and three‐dimensional conformal radiation therapy (3D‐CRT) plans

	Dose (Gy)			
	Mean		EUD	
Case	IMRT	3D‐CRT	IMRT	3D‐CRT
1	62.52	59.83	60.95	59.38
2	62.07	60.71	60.39	57.89
3	61.45	59.53	61.13	59.35
4	61.98	59.91	61.65	58.96
5	61.47	60.20	59.82	50.02
6	60.88	60.68	60.78	60.53
7	61.75	60.47	60.47	57.58
8	62.52	60.27	62.27	59.91
9	60.05	59.20	56.32	50.21
10	61.68	58.62	60.74	54.76
11	61.42	59.01	59.66	56.42
12	61.85	59.59	58.94	58.00
13	61.80	59.96	61.42	59.88
14	61.10	60.90	60.91	60.63
15	62.21	60.94	61.89	60.02
16	61.75	60.41	61.02	59.95
17	62.08	60.49	61.98	60.18
18	61.65	60.39	61.44	60.18
19	62.96	60.96	62.26	59.96
20	61.70	59.19	61.10	57.33
Average	61.74	60.06	60.76	58.06

**Table 3:** Percent normal brain volumes receiving doses equal to or higher than 18, 24, and 45 Gy with intensity‐modulated radiation therapy (IMRT) and three‐dimensional conformal radiation therapy (3D‐CRT) plans

	Volume (%)					
	≥18 Gy		≥24 Gy		≥45 Gy	
Case	IMRT	3D‐CRT	IMRT	3D‐CRT	IMRT	3D‐CRT
1	59.77	40.39	45.31	37.47	13.27	22.95
2	63.15	48.48	53.18	45.29	24.7	34.59
3	33.8	50.52	28.42	35.67	11.51	13.67
4	51.55	66.49	38.62	60.48	12.09	18.97
5	40.06	23.51	26.23	21.06	6.81	12.98
6	33.52	35.6	27.49	22.56	6.71	5.81
7	62.24	76.01	48.41	64.84	9.4	16.26
8	40.17	52.22	25.96	34.51	5.24	13.94
9	32.65	35.51	21.8	23.66	3.68	6.56
10	56.48	46.42	44.53	37.86	7	7.5
11	50.47	63.3	38.37	44.04	12.39	20.04
12	34.84	46.23	27.48	27.71	7.78	8.24
13	24.27	32.72	19.55	24.65	5.31	8.32
14	55.41	65.59	40.25	49.36	8.35	19.34
15	17.27	23.7	10.63	11.66	1.7	3.38
16	45.24	62.93	31.08	46.54	6.85	9.88
17	58.11	69.98	40.15	54.27	8.21	24.83
18	48.62	57.02	32.35	52.77	7.69	22.49
19	60.2	73.1	44.04	51.29	16.4	23.97
20	62.9	63.86	47.41	56.19	14.39	23.05
Mean	46.54	51.68	34.56	40.09	9.47	15.84
*p* Value	=0.059		=0.015		<0.0001	

**Table 4:** Percent normal brainstem volumes receiving doses equal to or higher than 45 Gy and 54 Gy with intensity‐modulated radiation therapy (IMRT) and three‐dimensional conformal radiation therapy (3D‐CRT) plans

	Volume (%)			
	≥45 Gy		≥54 Gy	
Case	IMRT	3D‐CRT	IMRT	3D‐CRT
1	3.39	5.02	0.3	0.13
2	20.62	3.46	0.06	0.04
3	2.14	4.27	0	0
4	15.07	40.84	0.78	10.16
5	0.05	0.01	0	0
6	0	23.82	0	0
7	43.24	30.96	25.98	0.81
8	13.82	27.07	0	0.47
9	38.25	50.24	18.43	16.62
10	38.55	39.21	0.5	0.28
11	29.89	70.74	4.37	41.27
12	40.11	63.32	24.2	35.03
13	0	0	0	0
14	66.33	82.71	49.43	64.79
15	0	4.92	0	0
16	32.77	53.97	0	0.06
17	40.16	64.94	12.7	23.14
18	13.62	20.55	5.67	9.92
19	2.13	14.25	0	0.05
20	53.02	56.11	26.79	40.03
Mean	22.66	32.82	8.46	12.14
*p* Value	0.004		0.165	

**Figure 2:**
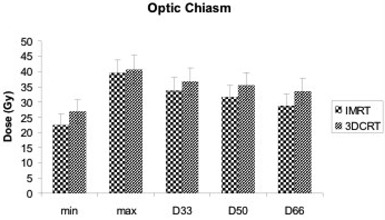
Minimum dose (min), maximum dose (max), and dose to 33% of organ volume ${(D}_{33})$, 50% of organ volume ${(D}_{50})$, and 66% of organ volume ${(D}_{66})$ for optic chiasm with intensity‐modulated radiation therapy (IMRT) and three‐dimensional conformal radiation therapy (3D‐CRT) plans.

**Figure 3:**
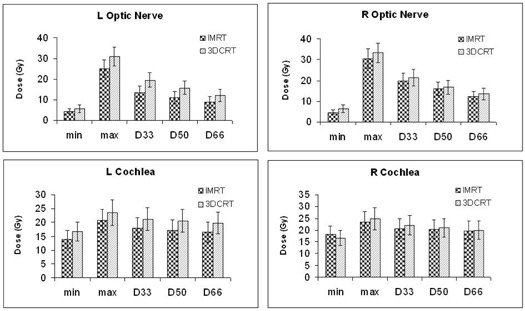
Minimum dose (min), maximum dose (max), and dose to 33% of organ volume ${(D}_{33})$, 50% of organ volume ${(D}_{50})$, and 66% of organ volume ${(D}_{66})$ for optic nerves and cochlea with intensity‐modulated radiation therapy (IMRT) and three‐dimensional conformal radiation therapy (3D‐CRT) plans.

The $D_{33}$, $D_{50}$, and $D_{66}$ for the optic chiasm (Fig. 2) was 33.77, 31.62, and 28.85 Gy respectively with IMRT and 36.67, 35.30, and 33.58 Gy with 3D‐CRT. Those differences were statistically significant for $D_{50}$ and $D_{66}\;(p\leq 0.026)$. Moreover, the percent volume of optic chiasm receiving more than 45 Gy was significantly reduced with IMRT by 30.40% (p=0.047). The $D_{33}$, $D_{50}$, and $D_{66}$ for the left optic nerve were significantly lower with IMRT (13.24, 10.96, and 8.92 Gy respectively) than with 3D‐CRT (19.56, 15.76, and 12.15, p≤0.05). Although IMRT also reduced the radiation doses to the right optic nerve and the cochlea, the differences were small and not statistically significant (Fig. 3).

**Figure 4:**
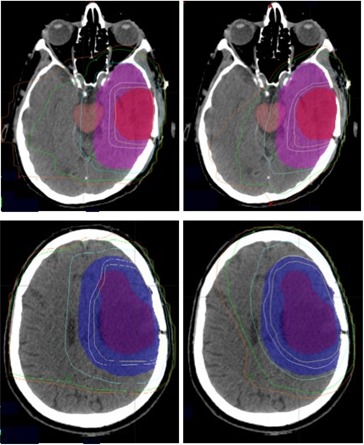
Isodose distribution in two patients, one with a left temporal lobe tumor and the other with a left frontoparietal tumor planned with three‐dimensional conformal radiation therapy (3D‐CRT, left) and intensity‐modulated radiation therapy (IMRT, right) techniques. For 3D‐CRT, both patients had anteroposterior and left lateral wedge paired fields. (The second patient had an additional right lateral field during the first 4500 cGy.) For IMRT, five oblique beams were used for both patients. From right to left, the isodose lines correspond to doses of 1800 cGy, 2400 cGy, 4500 cGy, 5643 cGy (95% of prescription dose), and 5940 cGy respectively.

Fig. 4 shows representative axial CT slices that demonstrate the isodose distributions of 3D‐CRT (left) and IMRT (right) plans in two patients, one with a left temporal lobe tumor and the other with a left frontoparietal tumor. The IMRT plan clearly minimizes exposure of normal brain, brainstem, and optic chiasm to higher radiation doses.

**Figure 5:**
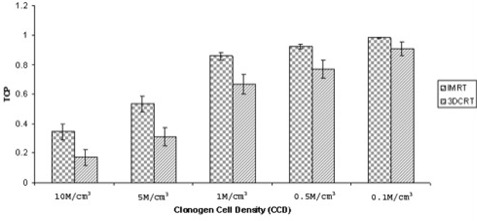
Comparison of tumor control probability (TCP) as a function of clonogen cell density (CCD) for intensity‐modulated radiation therapy (IMRT) and three‐dimensional conformal radiation therapy (3D‐CRT) plans. The CCDs are expressed in millions of cells (M) per cubic centimeter (cm^3^) of tumor volume.

As shown in Fig. 5, the TCPs were calculated for the IMRT and 3D‐CRT plans, and were found to be a function of CCD. Regardless of CCD, IMRT always resulted in TCP values that were superior to those calculated for 3D‐CRT. Higher CCD values resulted in poorer TCP. Specifically, for CCDs of 10, 5, 1, and 0.5 M/cm^3^, the corresponding TCPs were 34.55%, 53.50%, 86.04%, and 92.57% for IMRT and 17.17%, 31.24%, 66.75%, and 77.35% for 3D‐CRT—the differences all being statistically significant (p≤0.005). The best TCP values (98.43% for IMRT, and 90.86% for 3D‐CRT) were obtained at the lowest CCD of 0.1 M/cm^3^ (p=0.09).

**Figure 6:**
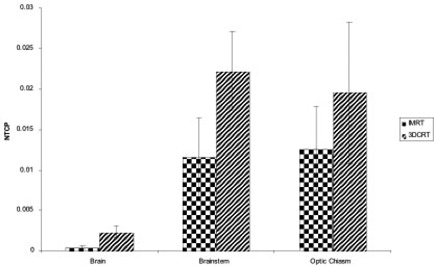
Normal‐tissue complication probability (NTCP) for brain, brainstem, and optic chiasm with intensity‐modulated radiation therapy (IMRT) and three‐dimensional conformal radiation therapy (3D‐CRT) plans.

Fig. 6 compares the NTCPs for brain, brainstem, and optic chiasm in the IMRT and 3D‐CRT plans. Specifically, IMRT significantly lowered the NTCP for brain and brainstem from 0.0023 with 3D‐CRT to 0.00043 (p=0.003) and from 0.02225 with 3D‐CRT to 0.01154 (p=0.033). For the optic chiasm, NTCP was reduced from 0.0196 with 3D‐CRT to 0.0126 with IMRT (p=0.067). Small NTCP reductions were also observed for the optic nerves and the cochlea, but those differences were not statistically significant.

## IV. DISCUSSION

Our results indicate that, as compared with 3D‐CRT, IMRT has the potential to increase the therapeutic window of radiation therapy for the treatment of high‐grade glioma. In the present study, IMRT improved tumor coverage and simultaneously reduced radiation dose to the normal brain, brainstem, and optic chiasm.

Data comparing IMRT to 3D‐CRT planning in brain gliomas are scarce. Studying a group of 5 patients, Chan et al. demonstrated that, as compared with 3D‐CRT, IMRT delivered higher doses (in excess of 10 Gy) to the gross tumor while respecting the same normal‐tissue constraints.^(23)^ In the present study, IMRT improved target coverage and dose homogeneity over 3D‐CRT by significantly reducing the percent target volume receiving less than 59.4 Gy from 22.59% to 3.3% and by increasing EUD by 4.7%. Moreover, IMRT also reduced the normal brain volume receiving doses of 18 Gy and 24 Gy or more by 10% and 14% respectively, and the volumes of brainstem and optic chiasm receiving more than 45 Gy by 31% and 30.4% respectively.

Our data are comparable to those reported by Narayana et al. In his study, IMRT significantly reduced the normal‐brain volume receiving doses of 18 Gy and 24 Gy or more by 7.5% and 8.3% respectively (p=0.01). In addition, the mean brainstem dose was also reduced by 7.4%.^(22)^

Clinicians encounter two major difficulties in planning the radiation for malignant gliomas. The first is associated with the inherent radioresistance and the treatment‐induced accelerated proliferation of these tumors.^(^ ^36^ ^,^ ^37^ ^)^ The second relates to the radiation tolerance of the surrounding normal brain.

Various radiation dose intensification regimens have been used to overcome radioresistance and accelerated repopulation. Unfortunately, these regimens have failed to show a significant benefit in most clinical trials.^(^ ^10^ ^,^ ^38^ ^)^ However, a few studies demonstrated an advantage in survival and local control in carefully selected patients with high‐grade glioma. Specifically, in a dose escalation trial by the Radiation Therapy Oncology Group, patients with glioblastoma multiforme treated to higher doses of 76.8 Gy or 81.6 Gy, at 1.2 Gy twice daily, had a slightly prolonged survival (11.6 months) as compared with patients treated with lower doses of 64.6 Gy or 72 Gy (10.2 months).^(39)^ In a phase II study using accelerated photon and proton therapy to a total dose of 90 cobalt Gy, overall survival was prolonged to 20 months, and all local failures but one occurred in volumes receiving doses of 70 Gy or less.^(40)^ Finally, some institutional studies have demonstrated a survival benefit (median survival: 16 – 23 months) with a brachytherapy or radiosurgery boost added to involved‐field radiation therapy in patients with smaller tumors.^(41)^ However, other studies indicate that this survival benefit may, at least in part, be attributable to selection bias.^(42)^

All of the above studies suffered from the inability of imaging technologies to accurately define tumor volumes. The extent of irradiation evolved from whole‐brain to involved‐field, given that radiation failures generally occur within 2 cm of the gross tumor volume defined by CT or MRI.^(7)^ However, recent developments in central nervous system imaging technology may further increase the ability to more precisely target areas at risk of failure.

Magnetic resonance spectroscopy (MRS) images normal‐brain or tumor metabolites to distinguish normal from malignant tissue or necrosis.^(^ ^43^ ^–^ ^45^ ^)^ Magnetic resonance perfusion uses differences in regional cerebral blood flow and vessel permeability to determine the likelihood of malignancy.^(46)^ Clinicopathologic correlations are underway to establish the utility of MRS or MR perfusion in grading gliomas.^(^ ^45^ ^,^ ^47^ ^–^ ^49^ ^)^ In addition, positron emission tomography techniques are being developed to map tumors for hypoxia, which induces further resistance to therapy.^(50)^

Currently, protocols are in progress to determine how to incorporate these new “functional imaging” technologies into the radiation treatment planning process.^(51)^ If gliomas can be accurately mapped, IMRT may provide further advantages because of its ability to target selected, more resistant parts within tumor with higher radiation doses without increasing the doses to normal tissues.^(^ ^52^ ^,^ ^53^ ^)^

Uncertainties in target volume definition may not only result in marginal misses of tumor, but also in unnecessarily overdosing normal brain. Delayed radiation injury is underestimated in patients with high‐grade glioma because of their limited life span and very high rate of local tumor progression. Nevertheless, higher radiation doses, fraction sizes above 2 Gy, large radiation volumes, and the use of chemotherapy have been shown to increase the risk of late radiation effects.^(^ ^54^ ^–^ ^56^ ^)^ Specifically, the risk of brain necrosis is 5% at 60 Gy.^(31)^ Radiation necrosis causes significant morbidity from persistent cerebral edema, and neurologic deficits may require steroid treatment or surgical management. Late radiation toxicity also includes deterioration of cognitive and visual function.^(^ ^31^ ^,^ ^57^ ^,^ ^58^ ^)^ Available data for long‐term survivors of high‐grade glioma indicate that dementia is common. Debilitation and cognitive decline have also been documented in patients receiving radiation for low‐grade glioma.^(^ ^3^ ^,^ ^58^ ^)^ Radiation therapy evidently poses a risk for delayed brain injury, and the superior dosimetric profile of IMRT may prove useful in minimizing treatment‐related morbidity in long‐term survivors.

As compared with 3D‐CRT, IMRT also results in superior TCP and NTCP. Specifically, TCP inversely correlates with CCD, being poorest at the highest clonogenic tumor cell concentration. The custom has been to assume CCD values of 10 M/cm^3^ for TCP calculations.^(^ ^24^ ^,^ ^25^ ^)^ In reality, however, the CCD for each individual tumor is unknown. In the present study, we used the ${\text{PTV}}_{\text{cd}}$ volumes for TCP calculations because the ${\text{PTV}}_{\text{cd}}$ is where the radiation dose is prescribed. However, the ${\text{PTV}}_{\text{cd}}$ includes areas of gross tumor volume and subclinical disease where clonogen cell concentrations may be quite different. In addition, we did not factor in hypoxia into our TCP calculations; otherwise, even lower TCP values would have resulted.^(^ ^59^ ^,^ ^60^ ^)^ Our results demonstrated that TCP increased from 34.55% at a CCD of 10 M/cm^3^ to 98.43% at a CCD of 0.1 M/cm^3^ for the IMRT group and from 17.17% to 90.86% for the 3D‐CRT group. These results correlate well with the poor clinical outcomes seen with standard adjuvant therapy and radiation doses of 60 Gy. Therefore, therapeutic interventions that achieve further cytoreduction—for example, radiation dose escalation with IMRT and the use of more effective systemic agents—are justified to improve biologic response in this disease.

New, targeted therapies may also improve outcomes in malignant glioma. As the understanding of tumor molecular behavior increases, prognostic markers are likely to be identified, permitting therapy to be successfully individualized based on specific molecular tumor targets.

Currently, a variety of agents that target cell membrane receptors, downstream signal transduction pathways, and angiogenesis are being tested alone or in combination with radiation therapy and chemotherapy in the setting of primary or progressive disease.^(^ ^16^ ^–^ ^18^ ^)^ High response rates of 63% and median survival in excess of 6 months were recently reported for patients with recurrent glioma treated with irinotecan and bevacizumab, a monoclonal antibody targeting vascular endothelial growth factor.^(16)^

## V. CONCLUSIONS

In the present study, target dose coverage was improved with IMRT planning as compared with 3D‐CRT planning, and dose to normal structures was concomitantly decreased. New diagnostic and therapeutic tools hold promise for improving outcomes in patients with high‐grade glioma. Clinical data and biologic response models suggest the need for further investigations of dose intensification in these tumors. Combining modern tumor imaging technology with IMRT will permit more accurate tumor definition and radiation dose intensification without increasing injury to normal brain and adjacent critical structures. Moreover, in the era of more effective systemic treatments and an increased number of long‐term survivors, the use of IMRT may minimize toxicity and improve quality of life.

## ACKNOWLEDGMENTS

The results of this study were presented at the 48th Annual Meeting of the American Society of Therapeutic Radiology and Oncology; November 5 – 9, 2006; Philadelphia, Pennsylvania.
